# An invisible soil acidification: Critical role of soil carbonate and its impact on heavy metal bioavailability

**DOI:** 10.1038/srep12735

**Published:** 2015-07-31

**Authors:** Cheng Wang, Wei Li, Zhongfang Yang, Yang Chen, Wenjing Shao, Junfeng Ji

**Affiliations:** 1 Key Laboratory of Surficial Geochemistry, Ministry of Education, School of Earth Sciences and Engineering, Nanjing University, Nanjing 210093, China; 2 Environmental Soil Chemistry Group, Delaware Environmental Institute and Department of Plant and Soil Sciences, University of Delaware, Newark, Delaware 19716, United States; 3 School of Earth Sciences and Resources, China University of Geosciences, Beijing 100083, China

## Abstract

It is well known that carbonates inhibit heavy metals transferring from soil to plants, yet the mechanism is poorly understood. Based on the Yangtze River delta area, we investigated bioaccumulation of Ni and Cd in winter wheat as affected by the presence of carbonates in soil. This study aimed to determine the mechanism through which soil carbonates restrict transport and plant uptake of heavy metals in the wheat cropping system. The results indicate that soil carbonates critically influenced heavy metal transfer from soil to plants and presented a tipping point. Wheat grains harvested from carbonates-depleted (due to severe leaching) soils showed Ni and Cd concentrations 2–3 times higher than those of the wheat grains from carbonates-containing soils. Correspondingly, the incidence of Ni or Cd contamination in the wheat grain samples increased by about three times. With the carbonate concentration >1% in soil, uptake and bioaccumulation of Ni and Cd by winter wheat was independent with the soil pH and carbonate content. The findings suggest that soil carbonates play a critical role in heavy metal transfer from soil to plants, implying that monitoring soil carbonate may be necessary in addition to soil pH for the evaluating soil quality and food safety.

Soil acidification, normally indicated by the pH decline of a certain soil, has recently received increasing attention due to its important impact on soil environmental quality, food security and human health^1^,^2^,^3^,^4^. Soil acidification often results in promoted leaching loss of beneficial elements and elevated mobilization and bioavailability of heavy metals^5^,^6^,^7^. The resistance of a soil to acidification is controlled by the buffering components such as carbonates and bicarbonates^8^ that are able to neutralize protons via chemical reactions. Liming with calcium carbonate is a widely-used agricultural practice for reducing soil acidity and decreasing bioavailability of heavy metals^9^,^10^,^11^.

The effect of carbonates on soil pH and heavy metal migration has been intensively studied^8^,^11^,^12^. It is well known that Ca^2+^ has a protective effect on metal uptake (especially on Cd^2+^) by the plants^8^. Nevertheless, the mechanisms through which liming materials affect the bioavailability of heavy metals in soil remain poorly understood. Field investigations in this area are rather limited. Under the background of global soil acidification, there is a need to unveil and quantify the relationships between soil acidification, carbonate leaching, and heavy metal transfer in the soil-plant system such that effective management practices can be developed to reduce the bioavailability of toxic trace metals.

The objectives of this study were to obtain a quantitative estimate of Ni and Cd accumulation in winter wheat (*T. aestivum*) as affected by soil carbonate leaching losses in field conditions and to address the interrelationships between soil pH, carbonates, and plant uptake of heavy metals. The Yangtze River delta in China was chosen as the study area. The area is an alluvial flood plain with concentrated industry and economy. Cultivation agriculture is also intensive and advanced in the region, with winter wheat as a staple crop in addition to rice. The delta serves as a transitional zone between the southern developed industrial area and the northern traditional agricultural area. Developed from marine strata (sedimentary rocks such as limestone, sandstone) and fluvial alluvial deposits, the soil in the delta region contained considerable amounts of carbonates in the past^13^. Compared with the acidic soils in the south and the alkaline soils in the north, the delta soils are generally neutral. Nevertheless, the region recently suffered from acid precipitation and significant soil acidification has been observed^3^,^14^. The average annual precipitation in the delta is about 2120 mm, and the average annual evaporation of water body is about 950–1100 mm. The pH of natural rainwater in the area is usually 5.6–6.5; however, acid rain (pH <5.5) occurred frequently in many localities. This led to a severe leaching loss of soil carbonates and concurrently heavy metal pollution of crops grown in the region, making the area an ideal experimental field to investigate the relationship between soil carbonate content, pH, acidification, and the bioavailability of heavy metals.

## Material and Methods

### Sampling

The study site was located at 30°00′N–33°20′N and 119°10′E–121°40′E in East China. According to Chinese soil taxonomy, soils within this site are primarily Anthrosols.

Prior to harvest of winter wheat in May, topsoil samples from 150 plots (200 m^2^ each) were collected from the top 20 cm of soil in 2010. A GPS (Global Positioning System) unit was used to locate the sampling sites. For each plot, 5 subsamples were gathered using a stainless steel trowel and mixed to form one composite sample. Corresponding wheat grain (*Triticum aestivum* L.) samples were collected from the plots. Five wheat grain sub-samples each from a 5 m × 5 m area were collected and combined into a single composite sample.

Soil samples were air-dried and sieved to <2 mm, aliquots were further ground to <0.074 mm for chemical analysis with the exception of pH measurement. The plant samples (grains) were carefully rinsed with deionized water, air-dried, and ground into fine particles (<0.074 mm). Oven-drying at 60 °C for 48 hrs was conducted prior to chemical analysis of the plant samples.

### Chemical Analysis

Soil pH was determined in a suspension of 1:2.5 soil to water ratio (w/v) using a pH meter (Model PHS-3C, Shanghai Precision and Scientific Instrument Co. Ltd., China). Soil bio-available Ni, Fe and Cd were extracted using the diethylenetriaminepenta-acetic acid (DTPA) method^15^, and analyzed by ICP-OES and ICP-MS, respectively. The soil samples were digested using the HCl-HNO_3_-HClO_4_-HF method^16^ prior to determination of the total heavy metal concentrations. Nickel concentrations were determined using inductively coupled plasma-optical emission spectrometry (ICP-OES, Thermo Element iCAP6000 (Radial), Cambridge, BZ, UK). Cadmium concentrations were determined with an inductively coupled plasma mass spectrometer (ICP-MS, Thermo Element X Series 2, Bremen, Germany). To determine the total concentrations of metals in wheat tissues, samples were digested using HNO_3_ and H_2_O_2_^7^. The concentrations of Ni and Cd were measured using ICP-MS.

Quality assurance and quality control (QA/QC) for metals in the soil and wheat samples were estimated by analyzing metal contents in blank and duplicate samples and Certified Reference Materials (GBW07402 and GBW07406 for soil, and GBW10011 and GBW10014 for wheat) approved by the General Administration of Quality Supervision, Inspection and Quarantine of the People’s Republic of China. Blank samples, duplicate samples, and Certified Reference Materials were included for every 20 samples in the chemical analysis process. The elemental recoveries and relative standard deviation (RSDs) for Certified Reference Materials were 94–106% and <5.0%, respectively.

### Soil Carbonate Analysis

The soil carbonate content was determined using a Fourier Transform Infrared Spectrophotometer (FTIR, Nicolet 6700, Thermo, USA) with the method by Ji *et al.* 17. Carbonate minerals have a characteristic absorption peak at a wave number 2520 cm^−1^, and this peak area can be used to quantify the amount of carbonates based on Equation 1. Analysis procedures are described in Supplementary Information 1.


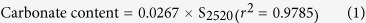


where S_2520_ is the area of the 2520 cm^−1^ peak.

### Statistical Analysis

Statistical analyses of data were performed using SPSS version 18 (SPSS Inc., USA). Pearson correlation coefficients were calculated to determine the relationship among parameters, and the data demonstrate a normal distribution. The spatial map of sampling sites was processed using the ESRI ArcGIS 9.3 program.

## Results

### Soil Carbonate and pH

**Figure 1:**
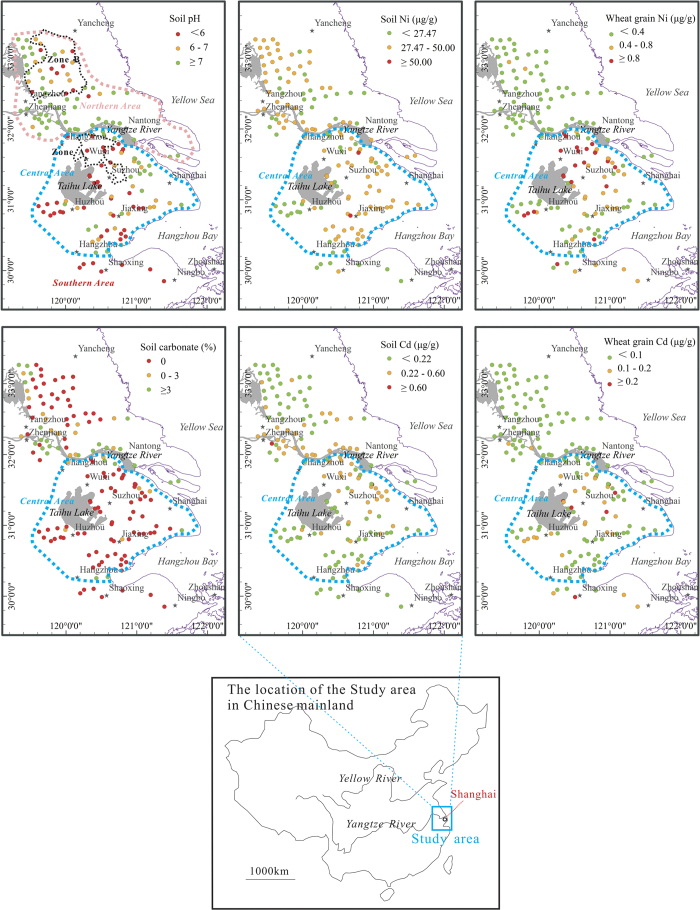
Spatial distributions of soil carbonate, pH, Ni and Cd and the corresponding wheat grain Ni and Cd. These maps were generated with software ArcGIS 9.5 (http://www.esri.com/software/arcgis/).

One hundred and fifty pairs of topsoil and corresponding wheat (*Triticum aestivum* L.) grain samples were collected from the Yangtze River delta area in 2010. Carbonate concentrations in the topsoil ranged from 0 to 18.5%, with a mean value of 1.4% (Fig. 1). About 60.7% of the samples did not contain carbonates, and these samples are considered as non-carbonate soil samples.

According to the analysis in this study, the pH of topsoil in the Yangtze River delta area ranged from 4.8 to 8.3, with a mean value of 6.7. Soil samples with pH <6.0 were primarily distributed in the southern portion of the Yangtze River delta region, and acidic-neutral soils were predominantly found along the Yangtze River and the northern portion of the delta (Fig. 1). At sites with soil pH <7.0, soil samples generally did not contain carbonates, while carbonate-containing soil samples demonstrated a pH >7.5.

### Ni and Cd Concentrations of Soils and Wheat Grain

The Ni concentration of topsoil in the Yangtze River delta area ranged from 10.2 μg g^−1^ to 55.2 μg g^−1^. The average Ni concentration was 30.17 μg g^−1^, close to the average Ni concentration of the overall Chinese topsoil (i.e., 29.3 μg g^−1^)^18^. The Topsoil Cd concentration ranged from 0.08 μg g^−1^ to 1.25 μg g^−1^, with an average of 0.22 μg g^−1^. The average Cd concentration in the study area was higher than the average level of the overall Chinese soil^18^. Most of the soil samples showed average Ni and Cd concentrations lower than the average values of the world soils^19^. Merely three samples contained Ni at concentrations greater than the average Ni composition of the upper continental crust^20^. According to the Chinese Soil Quality Standard^21^, only two of the soil samples had Ni or Cd concentrations exceeding the maximum permissible levels of agricultural soils (i.e., 50 μg g^−1^ for Ni and 0.6 μg g^−1^ for Cd). These results indicate that the topsoils of the investigated area were not heavily polluted by Ni or Cd.

Although the data suggest that the studied soils are generally safe in terms of total Cd and Ni concentrations, wheat grown at the site accumulated high levels of Ni and Cd. The concentration of Cd in the wheat grain ranged from 0.018 to 0.362 μg g^−1^, with a mean value of 0.075 μg g^−1^ (Fig. 1). The concentration of Ni was in the range of 0.070–1.480 μg g^−1^ and averaged at 0.383 μg g^−1^. These values were substantially higher than the routinely detected of wheat grains in other regions of China (e.g., 0.003–0.096 μg Cd g^−1^ 0.20–0.50 μg Ni g^−1^ in wheat grains from the Beijing area)^22^. According to Chinese maximum permissible concentrations of Cd (China Food Safety Standard Committee) in wheat grain (0.1 μg g^−1^)^23^, 22.7% of the wheat grain samples in this study were considered to be contaminated with Cd. Although only 14.0% of the samples exceeded the average Ni concentration of the world agricultural foods (0.8 μg g^−1^)^24^, the percentage would increase to approximately 34.0% if the safe Ni standard concentration (0.4 μg g^−1^) set by Chinese Food Safety Standard Committee was applied^22^.

In summary, the topsoil of the Yangtze River delta area was not high in total concentrations of Ni and Cd, but the produced wheat grains contained considerable amounts of Ni and Cd, with some exceeding the threshold limits.

### Comparison Wheat Accumulations of Ni and Cd on Ni and Cd Accumulations in Wheat Grain from Non-carbonate and Carbonate-containing Soils

Table 1 presents the pH and heavy metal total concentrations of carbonate-containing (CC) and non-carbonate (NC) soils and accumulations of heavy metals in wheat grown in the soils. The wheat grown in the NC soil showed higher Ni and Cd concentrations relative to that grown in the CC soil. The average Ni and Cd concentrations of wheat grains from the NC treatment were 0.51 μg g^−1^ (0.11–1.48 μg g^−1^) and 0.09 μg g^−1^ (0.02–0.36 μg g^−1^), respectively, significantly higher than those from the CC treatment (Ni: 0.19 μg g^−1^ (0.07–0.72 μg g^−1^); Cd: 0.06 μg g^–1^ (0.02–0.72 μg g^−1^)). However, the total Ni concentration of the NC soil (29.75 μg g^−1^ (10.20–55.20 μg g^−1^)) was close to that of the CC soil (30.82 μg g^−1^ (16.20–41.60 μg g^−1^)). The same phenomenon was observed for Cd, implying that the presence of carbonate plays a crucial role in wheat uptake and accumulation of Ni and Cd from soils.

**Figure 2:**
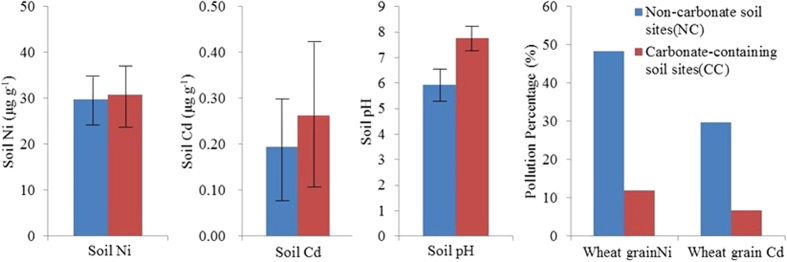
Comparison on soil Ni and Cd, pH and pollution percentages of Ni and Cd in wheat grain grown on the NC and CC soils. All the sampled sites were divided into two types: The corresponding wheat grain grown on the NC soil shows three times as much Ni and double the Cd concentrations relative to that of the CC soil, and the percentages of Ni and Cd contaminated wheat grain increase about three times, respectively.

Although wheat is not a crop that commonly accumulates heavy metals from soils^5^,^25^, after depletion of soil carbonates by leaching, the incidences of Ni and Cd contamination in wheat grain samples increased to 48.4% and 29.7%, respectively, approximately a 3-fold increase compared to the control CC treatment (Fig. 2). Evidently, the presence of soil carbonates suppressed the Ni and Cd accumulation in wheat. The reduced metal uptake from the CC soil was likely a result of the occurrence of carbonates that can inhibit metal mobilization by either maintaining a neutral to slightly alkaline soil environment or reducing free heavy metal ions in the soil solution.

**Figure 3:**
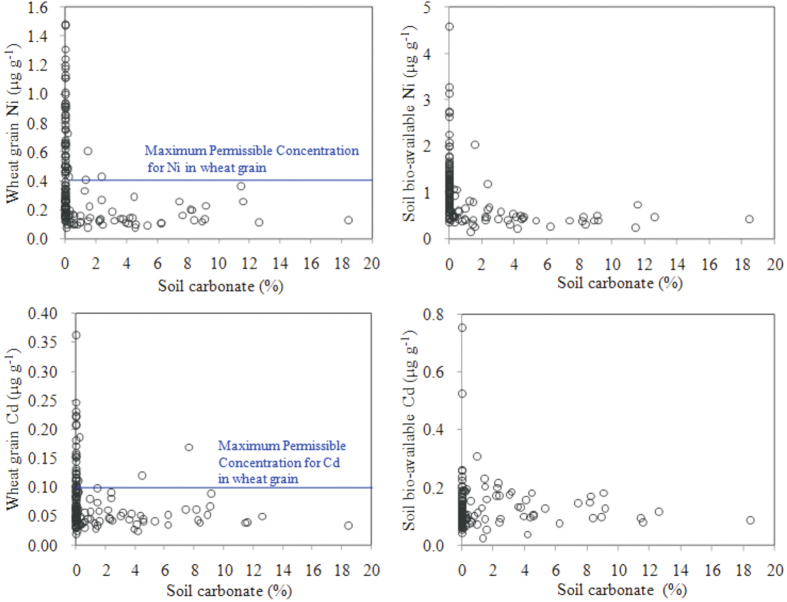
Ni and Cd concentrations in wheat grain and soil bioavailable Ni and Cd concentrations vs soil carbonate concentration (n = 150). Wheat grain Ni and Cd concentrations do not increase with decreasing soil carbonate concentration on the CC soil, but show a tipping point at a carbonate concentration near zero. Soil bio-available Ni and Cd concentrations significantly increased after carbonate loss.

It is noteworthy that neither Ni nor Cd concentrations in wheat grown in the CC soil correlated significantly with the soil carbonate concentration (Table 2; Fig. 3). The wheat grain concentrations of Ni and Cd remained generally constant as the soil carbonate concentration was above 1%. However, both metals presented a point of inflection when the soil carbonate concentration was under 1% (Fig. 3). As the soil carbonate concentration decreased to nearly zero, the variations in wheat grain Ni and Cd concentrations exhibited a significant increase, suggesting a high risk of Ni and Cd accumulation in wheat grown in carbonate-depleted soils.

## Discussion

Soils in the Yangtze River delta region were previously acidic in the southern portion and alkaline in the northern portion. The soils from the northern region and part of the central region often contained carbonate minerals derived from weathering of sedimentary rocks and alluvial deposits^13^. Historically, the existence of carbonate minerals maintained the topsoil with pH >7.0. The soil pH of the Yangtze River delta area was generally <7.0 in the south, >7.5 in the north, and 7.0–7.5 in the central, transitional delta zone^26^. However, a decrease in topsoil pH was observed for the study area over the past several decades, with recent pH typically <7.0 for the Yangtze River delta region (except the Yangtze River banks) in 2010 (Fig. 1).

**Figure 4:**
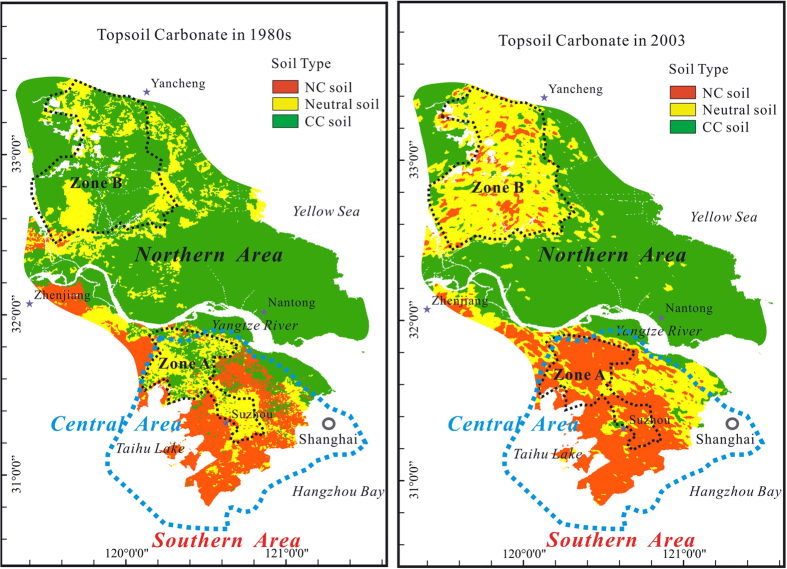
Comparison on soil carbonate of the Yangtze River delta area between 1980s and 2003. The map was generated with the software ArcGIS 9.5 and CorelDRAW 12. The carbonate concentration was estimated with soil pH cited from Reference 26. NC soil: non-carbonate soil with pH <6.5. CC soil: carbonate-containing soil with pH >7.5. Neutral soil generally has a pH value between 6.5 and 7.5.

Previous studies reported the occurrence of soil acidification in the Yangtze River delta^3^,^26^,^27^. Liu *et al.*^27^ estimated that the pH values of the topsoil from the medial region have declined 0.5–1.0 units during the past 30 years. This significant reduction is likely a result of atmospheric acid deposition derived from the accelerated industrial development after 1980s and excess application of nitrogen fertilizers in agricultural crop production^3^,^26^. One marked effect of soil acidification is the carbonate leaching loss from the topsoil as shown in Fig. 4, where it compares the topsoil carbonate contents of the Yangtze River delta area in 1980s and in 2003 (the data used in Fig. 4 were extracted from Wang *et al.*^26^). For instance, Zone A and Zone B in Fig. 4 show the areas that have suffered from serious acidification and carbonate leaching^26^. In contrast, these two regions lost most of their carbonates during the lapsed years.

According to a soil survey conducted before 1980s^13^, soils in the central and northern portion of the Yangtze River delta contained considerable amounts of carbonates. However, topsoil from most areas of the Yangtze River delta region (except the banks of the Yangtze River) showed no carbonates or rather low carbonate contents in 2010 (Fig. 1). Considering that spatial distribution of soil pH also declined during the same time period, it suggests that soil acidification resulted in carbonate loss from the topsoil horizon in the Yangtze River delta area.

**Figure 5:**
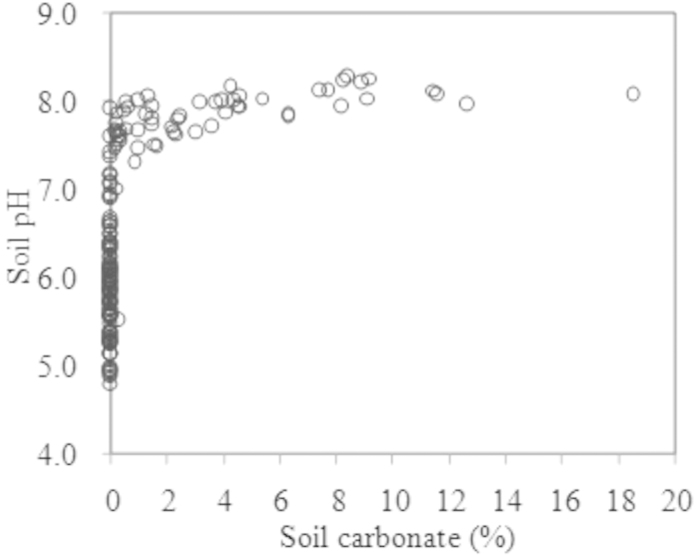
Corresponding relation between soil pH and carbonate concentration (n = 150). There is a crucial change between soil pH and carbonate concentration during the soil acidification process, i.e., soil pH shows a tipping point at the pH of 7 and carbonate concentration near zero.

The soil pH demonstrated a unique trend with the soil carbonate concentration (Fig. 5). When the soil carbonate content was above 2%, the soil pH remained relatively constant (~8). As carbonates were gradually leached to a concentration below 1% in soil, the soil pH sharply declined from 8 to 5 or lower. This provides evidence that soil carbonates act as a buffer and their adequate presence (e.g., >1% in soil) retards apparent acidification of a natural environment. Furthermore, this research demonstrates that a threshold carbonate content exists under field conditions, below which apparent acidification will dramatically increase. Depletion of soil carbonates should be regarded as invisible (or potential) acidification. Therefore, we argue that measuring soil pH only is inadequate for evaluating soil acidification and the soil carbonate content should be further considered.

In addition to buffering soil pH against acidification, soil carbonates are also important for sequestering heavy metals. For example, heavy metals bound to sulfides can be transformed to the carbonate phase in topsoil layers^28^. It is well-known that soil pH plays an important role in the speciation and stability of heavy metals associated with carbonate^29^. In the present study, as the topsoil was acidified from pH ≥7.8 (the CC soil) to pH = 6.0 (the NC soil), carbonates were completely leached out. As a result, heavy metals bound to carbonates were released into the soil solution phase, increasing their activities and bio-availability (Fig. 3). This enhanced the transfer of Ni and Cd from soils to plants, resulting in metal accumulation in wheat. This is confirmed by results showing higher Ni and Cd concentrations in wheat grown in the NC soils compared to in the CC soils, despite the comparable Ni and Cd concentrations for NC and CC soils.

Although it is well-known that decreases in soil pH can increase heavy metal transfer from soils to plants^6^,^7^, soil pH, bio-available Ni, and bio-available Cd did not correlate with wheat grain Ni or Cd concentration in the CC soils as observed in the present study. A significant correlation between pH and wheat grain Ni and Cd concentrations was observed only in the NC soils (Table 2). This indicates that the significant effect of soil pH on Ni and Cd transfer from soil to wheat only occurs in carbonate-depleted soils.

The above results demonstrate that a minimum soil carbonate content must be required for maintaining the soil pH buffering capacity. In this study, it was found that 1% carbonate, the value previously defined to differentiate between CC and NC soils, may be the minimum value for soil to buffer against acidification in the Yangtze River delta. By comparing Fig. 3 with Fig. 5, a similar relationship between soil carbonate content and bio-available metals was also observed, confirming that a threshold of carbonate content (i.e., 1%) exists for sequestration of heavy metals. We hypothesize that three processes may occur: 1) the initial acidification may cause carbonates in the topsoil to dissolve and be leached; however, soil pH does not change due to the neutralization of protons by carbonates; 2) as acidification further increases and the carbonate content drops to below the threshold value, the soil begins to lose its buffering capability and pH decreases sharply; and 3) heavy metals bound in mineral precipitates are released to the soil solution phase due to the significant reduction in pH, promoting plant uptake and tissue accumulation of the metals (e.g., Ni and Cd in wheat grains).

## Conclusion

The carbonate concentration of soils in the Yangtze River delta region has been noticeably decreasing due to human-introduced acidification over the past 30 years, promoting plant uptake and accumulation of heavy metals from the acidified soils. When soil carbonates were severely leached to a concentration <1% in soil, the grains of winter wheat grown in the acidified soils showed three times as much Ni and twice as much Cd concentration relative to the wheat grains harvested from carbonate-containing soils (i.e., soil carbonate >1%). The leaching of soil carbonate is the prelude of soil acidification and is an invisible threat to food safety as the significant facilitating effect of low pH on heavy metals transfer from soil to plants was observed only in carbonate-depleted soil.

## Additional Information

**How to cite this article**: Wang, C. *et al.* An invisible soil acidification: Critical role of soil carbonate and its impact on heavy metal bioavailability. *Sci. Rep.* **5**, 12735; doi: 10.1038/srep12735 (2015).

## Supplementary Material

Supplementary Information

## Figures and Tables

**Table 1:** pH, Ni and Cd concentrations of soil and wheat grain on the CC and NC soils.

	NC soil (n = 91)			CC soil (n = 59)		
	Mean	Max	Min	Mean	Max	Min
Soil pH	5.95	7.91	4.80	7.76	8.28	5.27
Soil Ni (μg g^−1^)	29.75	55.20	10.20	30.82	41.60	16.20
Soil Cd (μg g^−1^)	0.195	0.997	0.080	0.264	1.249	0.081
Wheat grain Ni (μg g^−1^)	0.51	1.48	0.11	0.19	0.72	0.07
Wheat grain Cd (μg g^−1^)	0.09	0.36	0.02	0.06	0.19	0.02
Carbonate (%)	0.00	0.00	0.00	3.65	18.46	0.09


**Table 2:** Pearson correlation coefficients between soil parameters and wheat grain Ni and Cd concentrations.

	All samples (n = 150)		CC soil (n = 59)^a^		NC soil (n = 91)^a^	
	Wheat grain Ni	Wheat grain Cd	Wheat grain Ni	Wheat grain Cd	Wheat grain Ni	Wheat grain Cd
Total Soil pH	−0.561**	−0.346**	−0.027	−0.029	−0.400**	−0.238*
Total Soil Ni	−0.032	−0.059	0.225	0.075	−0.045	−0.075
Total Soil Cd	0.018	0.347**	0.012	0.548**	0.243*	0.471**
Soil DTPA-Fe	0.578**	0.421**	0.307*	0.314*	0.380**	0.323**
Soil DTPA-Cd	0.099	0.509**	−0.020	0.301*	0.129	0.576**
Soil DTPA-Ni	0.521**	0.354**	0.131	0.067	0.395**	0.265*
Carbonate	−0.308**	−0.181*	−0.127	−0.065	/	/

^a^CC: carbonate-containing soil site, NC: non-carbonate soil site;

^**^Correlation is significant at the 0.01 level (2-tailed).

^*^Correlation is significant at the 0.05 level (2-tailed).
