# Supplementary material for: An invisible soil acidification: Critical role of soil carbonate and its impact on heavy metal bioavailability
Source: Sci Rep. 2015 Jul 31;5:12735. doi: 10.1038/srep12735 (PMC4521204; doi:10.1038/srep12735)
Supplement: Supplementary Information [file srep12735-s1.pdf]

Supplementary Information 1

An invisible soil acidification: Critical role of soil carbonate and its impact on metals' bioavailability

Cheng Wang[1], Wei Li[1,2], Zhongfang Yang[3], Yang Chen[1],Wenjing Shao[1], Junfeng Ji[1,*]

[1] Key Laboratory of Surficial Geochemistry, Ministry of Education, School of Earth Sciences and Engineering, Nanjing University, Nanjing 210093, China

[2] Environmental Soil Chemistry Group, Delaware Environmental Institute and Department of Plant and Soil Sciences, University of Delaware, Newark, Delaware 19716, United States

[3] School of Earth Sciences and Resources, China University of Geosciences, Beijing 100083, China

[*]Correspondence and requests for materials should be addressed to J.J. (email: jijunfeng@nju.edu.cn).

Supplementary Information 1

The analysis procedures of soil carbonate content determination using FTIR

Soil carbonate content was determined with the Fourier Transform Infrared Spectrophotometry (FTIR, Nicolet 6700, Thermo, USA), according to Ji et al[1]. The carbonate minerals have a characteristic absorption peak at a wave number 2520 cm^{-1},

and this peak is very sensitive in carbonate content change. It can identify carbonate content as less as 0.5% (Fig. S1). The detailed soil carbonate content analysis method is as follows:

Standard sample

The non-carbonate soil sample XM12 was used as the background, and different amount of $CaCO_3$ were added to make a series of standard samples with different carbonate contents that are listed in Table S1.

Collect FTIR data and determine of the area of the 2520cm^{-1} peak

For FTIR analysis, a 0.3 g of sample was ground to <2.5 μm with an agate mortar, thoroughly dried at 105 ℃, and placed in a cylindrical sample cup that was 12 mm in diameter and 3 mm deep. Samples were analyzed in a Thermo Nicolet 6700 FTIR with a diffuse reflectance attachment from wave numbers 4000–400(cm^{-1}) at Nanjing University. The sample chamber of the FTIR was kept in desiccated state, but was not evacuated, and the background was corrected to remove the effect of any remaining H_2O. Each analysis took about 2 min for gathering 128 spectra that were co-added to reduce noise. Data were recorded as percent reflectance relative to KBr, which was used as a standard to calibrate the machine. The area of the 2520cm^{-1} peak in spectra was measured using the program OMNIC 8.2 (Table S1).

Set up the estimated equation

The area of the 2520cm^{-1}peak to the carbonate content was correlated, and the fitted curve was determined (Fig. S2). The equation on estimating the carbonate content is:

$$\text{Carbonate content} = 0.0267 \times S_{2520}\ (r^2=0.9785) \qquad \text{(eq. 1)}$$

where S_{2520} is the area of the 2520cm^{-1} peak.

Determination of soil sample carbonates content

Soil sample was ground to <2.5 μm with an agate mortar, thoroughly dried at 105 ℃, and then analyzed using a Thermo Nicolet 6700 FTIR according to the method described in 2). The carbonate content was calculated using eq.1.

References

1. Ji, J., Ge, Y., Balsam, W., Damuth, J. & Chen, J. Rapid identification of dolomite using a Fourier Transform Infrared Spectrophotometer (FTIR): A fast method for identifying Heinrich events in IODP Site U1308. *Mar. Geol.* **258**,60–68(2009).

Table S1

Table S1 Carbonate concentration gradient of standard samples and the corresponding area of the 2520cm^{-1} peak

Sample No.	Carbonate content (%)	Area of the 2520cm^{-1} peak
SB0	0	0
SB1	0.50	13.259
SB2	0.75	30.392
SB3	1.00	42.487
SB4	1.25	50.451
SB5	1.50	50.690
SB6	1.75	74.049
SB7	2.00	70.396
SB8	3.00	114.131
SB9	4.00	148.024
SB10	5.00	188.972
SB11	6.00	224.825
SB12	8.00	280.280
SB13	10.00	399.838
SB14[a]	1.00	51.081
SB15[b]	2.00	67.215

[a], the repetition of SB3; [b], the repetition of SB7.

Supplementary Figure caption

Fig. S1 Sectional Fourier Transform Infrared Spectrophotometry of standard samples. Carbonate minerals have a characteristic absorption peak at a wave number of 2520 cm^{-1}, and the intensity shows a relationship with carbonate content.

Fig. S2 Correlation between the carbonate content of standard samples and the area of the 2520 cm^{-1} peak.

Fig. S1

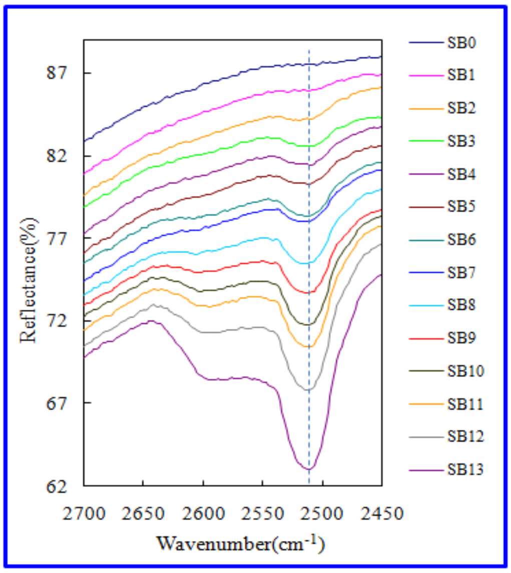
87
82
77
72
67
62
Reflectance(%)
2700
2650
2600
2550
2500
2450
Wavenumber(cm-1)
SB0
SB1
SB2
SB3
SB4
SB5
SB6
SB7
SB8
SB9
SB10
SB11
SB12
SB13

Fig. S2

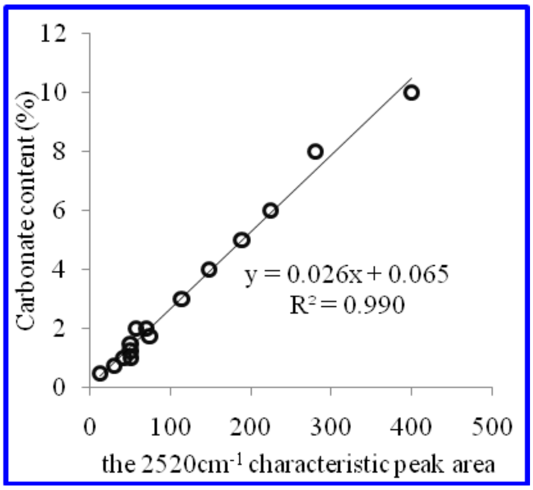
Carbonate content (%)
the 2520cm-1 characteristic peak area
y = 0.026x + 0.065
R2 = 0.990
0
2
4
6
8
10
12
0
100
200
300
400
500
